# Pictilisib Enhances the Antitumor Effect of Doxorubicin and Prevents Tumor-Mediated Bone Destruction by Blockade of PI3K/AKT Pathway

**DOI:** 10.3389/fonc.2020.615146

**Published:** 2021-02-15

**Authors:** Chao Liang, Xijiao Yu, Naping Xiong, Zhichang Zhang, Zhenyu Sun, Yang Dong

**Affiliations:** ^1^ Department of Orthopedics, Shanghai Jiao Tong University Affiliated Sixth People’s Hospital, Shanghai, China; ^2^ Department of Endodontics, Jinan Stomatological Hospital, Jinan, China; ^3^ School of Life Science and Technology, ShanghaiTech University, Shanghai, China

**Keywords:** pictilisib, osteosarcoma, osteoclasts, targeted therapy, PI3K inhibitor

## Abstract

Despite advances in neoadjuvant chemotherapy, outcomes for patients with osteosarcoma resistant to first-line chemotherapy have been dismal for decades. There is thus an urgent need to develop novel targeted drugs to effectively treat refractory osteosarcoma. Dysregulation in the PI3K/AKT pathway has been observed during the development of osteosarcoma. Herein, we first evaluated p-AKT (Ser473) expression levels in osteosarcoma tissue using high-throughput tissue microarrays. Then, we demonstrated the role of pictilisib, a novel potent PI3K inhibitor, in osteosarcoma and related osteolysis. Functional studies of pictilisib in osteosarcoma cell lines and bone marrow-derived macrophages were performed *in vitro*. Patient-derived xenografts and orthotopic mouse models were used to assess the effects of pictilisib *in vivo*. The results showed that positive p-AKT expression levels after neoadjuvant chemotherapy were significantly associated with tumor cell necrosis rate. Pictilisib effectively inhibited the proliferation of osteosarcoma through G0/G1-S phase cell cycle arrest, and enhanced the sensitivity of osteosarcoma to doxorubicin, although it failed to induce cell apoptosis alone. In addition, pictilisib inhibited differentiation of osteoclasts and bone resorption *in vitro* and tumor-related osteolysis *in vivo via* inhibition of the PI3K/AKT/GSK3*β* and NF-*κ*B pathways. Pictilisib combined with conventional chemotherapy drugs represents a potential treatment strategy to suppress tumor growth and bone destruction in p-AKT-positive patients.

## Introduction

Osteosarcoma (OS) is the most frequent primary malignant bone tumor and occurs mainly in the young (1, 2). Currently, the standard therapy for newly diagnosed OS consists of four to six cycles of neoadjuvant chemotherapy with cisplatin, doxorubicin (DOX), methotrexate, and ifosfamide after biopsy. With this combination of drugs, the 5-year survival rate in non-metastatic OS patients has increased to 50–70%. However, the survival rate has not improved since the mid-1980s owing to a lack of targeted drugs (3, 4). Accumulating evidence indicates that activation of the PI3K/AKT pathway has a critical oncogenic role in the initiation and progression of OS (5). Enrichment of mutated genes in the PI3K/AKT pathway in the early and late stages of OS has been demonstrated by next-generation sequencing (6); alterations of genes in this pathway were detected in 24% of OS patients (7). Activation of the PI3K/AKT pathway has also been observed in the majority of OS cell lines by detecting kinome and mRNA expression profiling (8). Moreover, research suggests that the PI3K/AKT pathway contributes to multiple pathological processes of OS, including tumorigenesis, proliferation, invasion, cell cycle progression, lung metastasis, and chemoresistance (5). Ji et al. found that the expression of p-AKT (Ser473) was negatively correlated with tumor cell necrosis rate (TCNR) after chemotherapy (9). Thus, the PI3K/AKT signaling pathway is a potential source of therapeutic targets.

OS is an osteoblastic tumor, but many studies have shown that osteoclasts have a vital role in its progression (10–12). OS tumor cells originating from osteoblasts produce receptor activator of nuclear factor kappa B ligand (RANKL), which regulates the differentiation of bone marrow-derived macrophages (BMMs) into bone-resorbing osteoclasts expressing activator of nuclear factor kappa B (RANK). OS-activated osteoclasts continuously destroy mineralized bone matrix, resulting in the release of growth factors including transforming growth factor-*β* (TGF-*β*), insulin like growth factor-1 (IGF-1), fibroblast growth factor (FGF), and bone morphogenetic protein (BMP), and providing a niche microenvironment for OS cells (11). Based on evidence from bone metastasis, a “vicious cycle” involving osteoclasts, osteoblasts, and sarcoma cells has been hypothesized to occur during the development of OS. In a recent study by Francois et al., overexpression of osteoprotegerin, a soluble decoy receptor, in a mouse model of OS did not directly affect proliferation of tumor cells but indirectly decreased tumor growth by blocking the vicious cycle of tumor cell proliferation and bone resorption (13). The formation of osteoclasts is mainly regulated by RANKL and macrophage-colony stimulating factor (M-CSF), both of which initiate the activation of various downstream pathways including the PI3K/AKT and NF-*к*B signaling pathways. Thus, osteoclasts could be exploited as therapeutic targets (14).

With a greater understanding of the pathogenesis, progression, and microenvironment of OS at a molecular level, targeting the PI3K/AKT pathway may be a promising therapy for OS. The most direct approach to blocking the PI3K/AKT pathway is to target PI3K itself. The catalytic PI3K family consists of three classes (I–III), of which class I PI3Ks are the most relevant to cancers owing to their role in regulating cell proliferation and tumorigenesis (15). Inactivated PI3K cannot convert its substrate phosphatidylinositol 4,5-biphosphate into phosphatidylinositol 3,4,5-triphosphate. Thus, the downstream effector AKT is not phosphorylated. Pictilisib, also known as GDC-0941, inhibits all four class I PI3K p110 isoforms (16, 17) and has been shown to have significant efficacy in preclinical breast cancer and advanced solid tumors, with tolerable side effects (18–20).

In the present study, we investigated the ability of pictilisib to decrease proliferation and related osteolysis in OS cell lines *in vitro* and in two mouse models *in vivo*.

## Materials and Methods

### Human Osteosarcoma Tissue Microarray

A total of 60 tumor samples from 31 individual OS patients at Shanghai Sixth People’s Hospital between 2014 and 2018 were used to construct a high-throughput tissue microarray. These tissue blocks consisted of 29 biopsy samples, 31 resection samples obtained after chemotherapy, and five normal bone marrow tissue samples from patients with OS. Related clinical information was collected for all participants, including sex, age, location, Enneking stage, and chemotherapy response. Five-millimeter paraffin-embedded tissue section slides for tissue microarray were baked for 2 h at 60°C, deparaffinized in xylene, and then transferred through graded ethanol for rehydration. After heat-induced epitope retrieval, the endogenous peroxidase was quenched with 3% hydrogen peroxide. Proteins were incubated with rabbit anti-human p-AKT (Ser473) antibody (#4060, 1:100, Cell Signaling Technology, USA) at 4°C overnight. We next evaluated the expression of p-AKT (Ser473) using immunohistochemistry (IHC). The immunostaining density of p-AKT was classified as follows: 0, no staining; 1+, weak staining; 2+, moderate staining; and 3+, intense staining. No specific staining with p-AKT was observed in ordinary bone tissue. The project was approved by the ethics committee of Shanghai Sixth People’s Hospital.

### Drugs, Cell Lines, and Culture Conditions

Pictilisib (C_23_H_27_N_7_O_3_S_2_; MW, 513.64; purity, ≥99%) Doxorubicin hydrochloride (C27H29NO11*HCl; MW, 579.99; purity, 99.37%), and dimethyl sulfoxide (DMSO) were purchased from TargetMol (Shanghai, China) and stored at −20°C. Four OS cell lines (MG-63, U2OS, Saos2, and 143B) were cultured in Dulbecco’s modified Eagle’s medium (DMEM) supplemented with 10% fetal bovine serum (FBS) and 1% penicillin/streptomycin. Primary BMMs were isolated from C57BL/6 mice and cultured in *α*-modified Eagle’s medium supplemented with 1% penicillin/streptomycin, 10% FBS, and 30 ng/ml M-CSF at 37°C and 5% CO_2_ (21). Recombinant murine M-CSF and RANKL were obtained from R&D Systems (Minneapolis, MN, USA).

### Cell Viability Assay

The cell viability assay was performed as previously described (22). BMMs and OS cells were seeded in 96-well plates in DMEM (1 × 10^3^cells/well) in triplicate. Half-maximal inhibitory concentration (IC_50_) values were determined using a Cell Counting Kit-8 (Dojindo, Japan) on OS cell lines after treatment with escalating doses of pictilisib (from 0 to 10 μM) at time points from 24 to 72 h. Absorbance was measured at 450 nm on an ELx800 microplate reader (Bio-Tek Instruments, USA). The IC_50_ of pictilisib was calculated using the Prism v.5.0c software (GraphPad, San Diego, CA).

### Cell Cycle Analysis

Tumor cells were seeded in six-well dishes and incubated with a range of concentrations of pictilisib (from 0 to 5 μM) for 24 h. Floating and adherent cells were collected by trypsinization and washed with phosphate-buffered saline (PBS). Cells were incubated in 70% ethanol at −4°C overnight, treated with RNase A, then stained with propidium iodide (PI). Cell cycle distribution was determined using flow cytometry (CytoFLEX, Beckman Coulter, USA) and DNA cell cycle analysis software (ModFit LT, USA).

### Detection of Apoptotic Cells

Apoptosis of MG-63 and U2OS cells treated with pictilisib and/or DOX at various concentrations was assessed using a FITC Annexin V Apoptosis Detection Kit I (Becton Dickinson, USA) according to the manufacturer’s instructions. Treated cells were resuspended at a density of 1 × 10^6^ cells/ml in 100 μl binding buffer, then incubated with 5 μl PI and 5 μl Annexin V-FITC for 15 min at room temperature. Detection of apoptotic cells was performed using a flow cytometer (CytoFLEX, Beckman Coulter, USA).

### Osteoclast Differentiation and Resorptive Function Assay *In Vitro*

To assess osteoclast differentiation, BMMs were seeded at a density of 1 × 10^4^ cells/well in 96-well plates, stimulated with 30 ng/ml M-CSF and 50 ng/ml RANKL, and treated with the appropriate concentrations of pictilisib for approximately 7 days. Fresh media containing these two factors were changed every other day until multinucleated osteoclasts had formed in the RANKL-only control wells. After fixed with 4% paraformaldehyde for 30 min, cells were stained using a tartrate-resistant acid phosphatase (TRAP) kit. Digital images were captured with an optical light microscope (Olympus, Tokyo, Japan). The ImageJ software (NIH, Bethesda, MD, USA) was used to analyze the numbers and area of TRAP-positive osteoclasts with at least three nuclei.

For the bone resorption assay, BMMs were seeded in six-well plates and cultured with 30 ng/ml M-CSF and 50 ng/ml RANKL until small osteoclasts were observed. Then, the cells were reseeded in phosphate-coated Osteo Assay Stripwell Plates (Corning, NY, USA) in triplicate. Cells were incubated in media containing various concentrations of pictilisib (0, 0.5, 1, 2 μM) together with 30 ng/ml M-CSF and 50 ng/ml RANKL until day 7, when mature osteoclasts were observed. Osteoclasts were removed using 10% sodium hypochlorite solution. Subsequently, resorption pits were imaged under a light microscope (Leica, Wetzlar, Germany). The resorption pit area was analyzed with ImageJ software (NIH, Bethesda, MD, USA).

### Western Blotting

Treated cells were lysed with radio-immunoprecipitation assay and a phosphatase inhibitor (Sigma, MO, USA). Total protein extracts were separated by sodium dodecyl polyacrylamide gel electrophoresis and then transferred to polyvinylidene fluoride membranes for western blotting. The membranes were blocked with 5% skimmed milk for 1 h at room temperature, incubated with primary antibody (1:1,000) overnight at 4°C, and then with secondary antibodies (1:5,000) for 1 h. Finally, membranes were developed using an enhanced chemiluminescence substrate. Detailed information about antibodies is available in the supplemental files.

### Establishment of Patient-Derived Xenograft Model

Four-week-old female BALB/c nude mice were purchased from Shanghai Laboratory Animal Research Center (Shanghai, China). Three fresh surgical specimens with p-AKT-positive staining were obtained from different individuals with OS and cut into 1–3mm^3^ fragments under sterile conditions. Subsequently, they were implanted subcutaneously into three nude mice within 2 h. Only one tumor specimen, from a 15 year-old girl with chemotherapy-resistant OS, was successfully used to generate an experimental patient-derived xenograft (PDX) model. When the subcutaneously implanted tumors grew to approximately 1,000 mm^3^, they were dissected and passaged to other mice in preparation for subsequent studies. When tumors grew to approximately 100 mm^3^, they were randomly separated into four groups (n = 5) and treated with PBS [intragastric (i.g.) administration, every other day]; 50 mg/kg pictilisib (i.g. administration, every other day); 2 mg/kg DOX [2-day continuous intraperitoneal (i.p.) injection per week]; or 50 mg/kg pictilisib and 2 mg/kg DOX (i.g. administration, every other day and 2-day continuous i.p. injection per week). Tumor volumes were calculated every 7 days by the following standard formula: (length × width^2^)/2. After 28 days of treatment, all the mice were sacrificed, and the tumors were harvested and weighed. The animal study was approved by the Animal Ethics Committee of Shanghai Sixth People’s Hospital (Shanghai, China).

### Cell-Derived Orthotopic Mouse Model

An orthotopic mouse model was established using 143B-luc to evaluate the effects of pictilisib on tumor progression and bone absorption *in vivo*. Initially, 1 × 10^6^ cells were suspended in 100 μl BD Matrigel TM Matrix (Becton, USA) and subcutaneously injected into the back of one mouse. Three weeks later, the tumor sample was cut into 1 × 1 × 1 mm^3^ pieces. The left posterior limb of each nude mouse, anesthetized by chloral hydrate, was cut in the proximal part of the tibia, and one tumor fragment was inserted on the surface of tibia. After 2 weeks, the mice were randomly divided into four groups (n = 5) and treated as follows: 143B with PBS (i.g. administration), 143B with pictilisib (50 mg/kg, i.g. administration, every other day), 143B with pictilisib (100 mg/kg, i.g. administration, every other day), or 143B with pictilisib (50 mg/kg, i.g. administration, every other day) and DOX (2 mg/kg, 2-day continuous i.p. injection per week). Body weight of mice was measured at 1-week intervals for 4 weeks. *In vivo* bioluminescence imaging was performed to observe the progression of tumors at days 0, 14, and 28. After sacrifice, the left posterior limb of each nude mouse in each group was weighed.

### Micro-Scanning and Radiological Analyses

Destruction of fixed tibiae mediated by tumors was analyzed using a high-resolution micro-CT scanner (μCT-100; SCANCO, Bröttisellen, Switzerland). Parameters were set to an isometric resolution of 1–10 mm and X-ray energy settings of 70 kV and 200 μA. After reconstruction, the bone volume to total volume (BV/TV) ratio was measured in the region of interest.

### Immunohistochemistry, Ki67, and Hematoxylin and Eosin Staining

Fresh tumor samples and primary organs were fixed in 10% formalin and embedded in paraffin before sectioning and staining. Tumor specimens were stained with anti-human p-AKT (Ser473) antibody (#4060, 1:100, Cell Signaling Technology, USA) and anti-human cleaved caspase-3 (Asp175) (#9661, 1:400, Cell Signaling Technology, USA) antibody. Ki67 staining was performed to evaluate the proliferation of tumors with anti-ki-67 antibody (1 μg/ml, Abcam, Cambridge, MA). H&E staining was performed to assess the toxic effects of pictilisib on the heart, lung, liver, and kidney.

### Statistical Analysis

Statistical analysis was performed using SPSS version 18.0 software (IBM Corporation, Chicago). Data are shown as the mean ± SD of three independent experiments. Differences between experimental and control groups were calculated by two-tailed student’s t-tests. Correlations between p-AKT (Ser473) and expression levels and pathological features of patients with OS were analyzed by both χ^2^-test and student’s t-test. P <0.05 was regarded as significant for all statistical analyses.

## Results

### p-AKT Is Partly Positive in Human Osteosarcoma Tissues and Negatively Correlated With Tumor Cell Necrosis Rate After Chemotherapy

**Figure 1:**
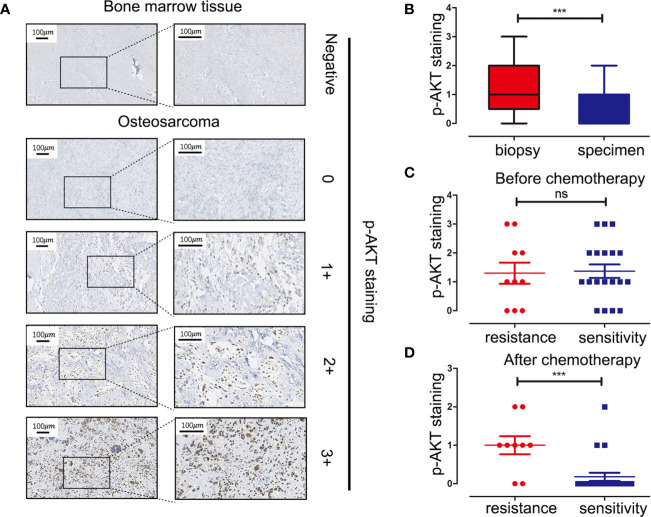
Immunohistochemistry staining of p-AKT (Ser473) in human OS tissue microarray and correlations between expression levels of p-AKT and drug resistance at different stages. **(A)** Representative images of different intensities of p-AKT staining in tissue microarray: 0, no staining; 1+, weak staining; 2+, moderate staining; 3+, intense staining. Bone marrow tissue was used as a negative control. **(B)** Comparison of p-AKT staining scores in biopsy and OS specimens. **(C)** Comparison of p-AKT staining scores between chemotherapy-resistant and chemotherapy-sensitive groups in biopsy samples **(D)** Comparison of p-AKT staining scores in post-chemotherapy specimens between chemotherapy-resistant and chemotherapy-sensitive groups ***P < 0.001; ns, no significance.

Tissue microarray analysis with 60 OS samples and five normal bone marrow samples was conducted to assess the expression levels of p-AKT (Ser473) using IHC. Clinical information of OS patients is shown in **Table 1**. Positive staining for p-AKT proteins manifested as purple particles in the cytoplasm, ranging from no staining (0, 28/60, 46.7%), weak staining (1+, 17/60, 28.3%), and moderate staining (2+, 10/60, 16.7%) to intense staining (3+, 5/60, 8.3%). Normal bone marrow tissue was used as a negative control group with no staining (**Figure 1A**). Subsequently, the correlations between p-AKT (Ser473) expression levels in biopsy samples and specimens after chemotherapy were analyzed. The p-AKT-positive rate in biopsy samples was significantly higher than that in specimens after chemotherapy (75.9 *vs* 32.2%, P = 0.002) (**Figure 1B**). Our results also confirmed that p-AKT (Ser473) had a significant correlation with TCNR after chemotherapy (χ^2^ = 11.487, P = 0.002) (**Figure 1D**), that is, positive p-AKT after chemotherapy was considered to be a sign of poor response to chemotherapy (TCNR <90%). By contrast, there was no significant difference between expression levels of p-AKT in biopsy samples and TCNR (χ^2^ = 0.758, P = 0.954) (**Figure 1C**) (**Table 2**).

**Table 1:** Demographic data for patients with OS.

Variable	Number
Average age at diagnosis (years)	13.56 ± 3.54
Gender	
Male	17
Female	14
Location	
Upper limb	11
Lower limb	18
Pelvis	2
Enneking stage	
II A	2
II B	25
III	4
Chemotherapy response	
Good response^a^	22
Poor response^b^	9

^a^Good response: TCNR ≥ 90%; ^b^Poor response: TCNR < 90%.

**Table 2:** Correlations between p-AKT (Ser473) expression level and chemotherapy response.

		Cases	p-AKT (Ser473) staining intensity score				χ^2^-value	P-value
			0	1+	2+	3+		
Stage	Biopsy^a^	29	7	10	7	5	16.368	0.002
	Specimen^a^	31	21	7	3	0
Biopsy	Sensitivity^b^	19	4	7	5	3	0.758	0.954
	Resistance^b^	10	3	3	2	2
Specimen	Sensitivity	22	19	2	1	0	11.487	0.002
	Resistance	9	2	5	2	0

^a^Biopsy: OS tissue before chemotherapy; ^a^Specimen: OS tissue after chemotherapy; ^b^Sensitivity: good response or TCNR ≥ 90%; ^b^Resistance: poor response or TCNR < 90%.

### Inhibitory Effect of Pictilisib Against Osteosarcoma Depends on the Induction of Cell Cycle Arrest, and Pictilisib Enhances the Sensitivity to Chemotherapy

**Figure 2:**
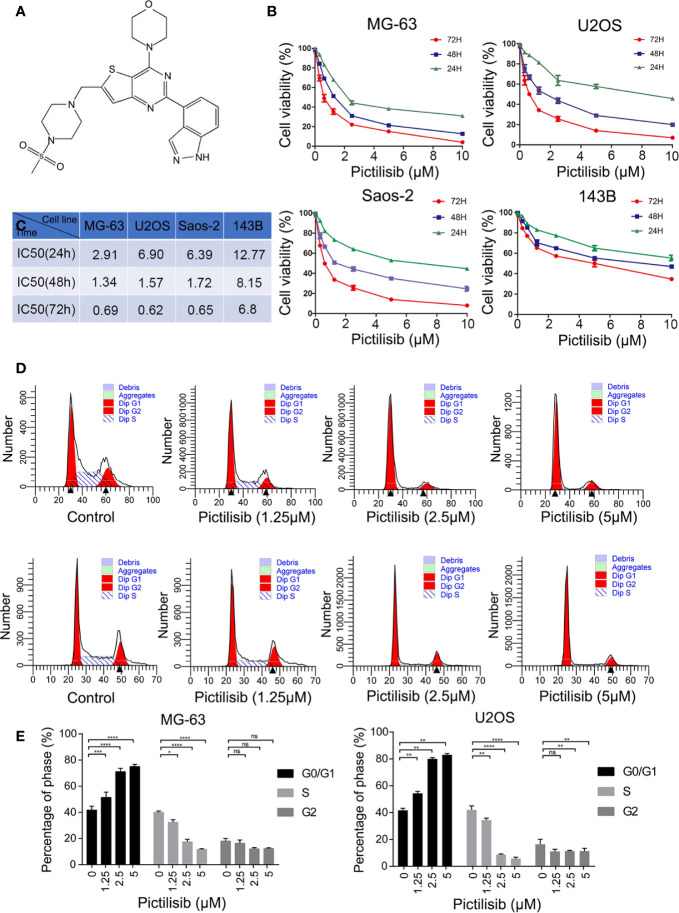
Pictilisib inhibited cell proliferation by inducing cell cycle arrest. **(A)** Chemical structure of pictilisib. **(B, C)** OS cell lines were treated with pictilisib at the indicated concentrations for 24, 48, and 72 h. IC_50_ values for the four OS cell lines were determined by CCK8 assays and are reported in the table. **(D, E)** Cells treated with pictilisib at the indicated concentrations for 24 h were stained with PI for flow cytometric analysis and analyzed by ModFit. Percentages of G0/G1, S, and G2 phase cells after treatment with the indicated concentrations of pictilisib. *P < 0.05, **P < 0.01, ***P < 0.001, ****P < 0.0001, ns, no significance.

**Figure 3:**
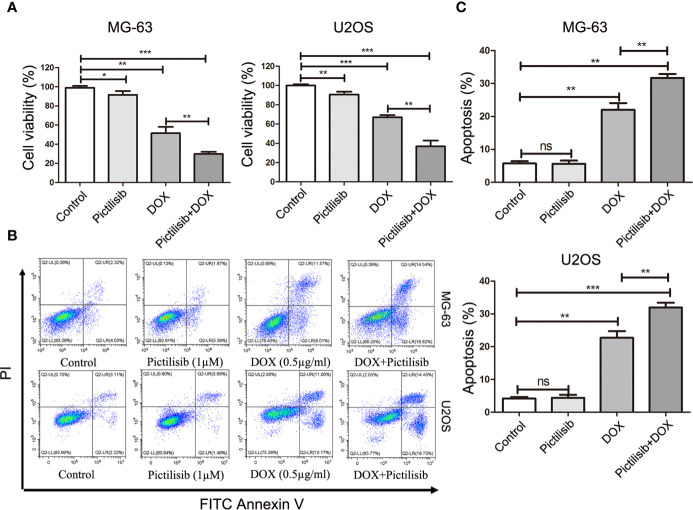
Low-dose pictilisib failed to induce cell apoptosis but enhanced sensitivity of OS cells to DOX. **(A)** Cells were treated with 1 μM pictilisib with or without 0.5 μg/ml DOX, and CCK8 assays were performed to evaluate cell viability. **(B, C)** Percentages of apoptotic cells treated with 1 μM pictilisib with or without 0.5 μg/ml DOX for 24 h were detected by using a flow cytometry. *P < 0.05, **P < 0.01, ***P < 0.001, ns: no significance.

To determine the effects of pictilisib (**Figure 2A**) on cultured OS cells, a CCK8 assay was performed on a panel of OS cell lines (MG-63, U2OS, Saos2, and 143B). The results showed that pictilisib dose-dependently inhibited OS cell proliferation for 24, 48, and 72 h (**Figure 2B**). At least 48 h was required for pictilisib to exert a significant inhibitory effect on OS cells, and MG-63 cells showed a strong response with the lowest IC_50_. Among the four OS cell lines, 143B was the least sensitive to pictilisib (**Figure 2C**). Next, cell cycle and apoptosis assays were performed to investigate how pictilisib inhibited proliferation of OS cells. We found that the majority of tumor cells were arrested in G0/G1-S phase by stepwise concentrations of pictilisib, in contrast to untreated cells (**Figures 2D, E** **)**. The apoptosis assays revealed no significant difference over a range of concentrations of pictilisib (0–2 μM) (**Supplemental Figures 1A, B** **)**. Thus, pictilisib exerted its inhibitory effect against OS *via* cell cycle arrest rather than induction of apoptosis. Considering the correlation between p-AKT expression levels and chemotherapy resistance in OS, we investigated whether pictilisib sensitized OS cells to chemotherapy. The results of the CCK8 assays indicated that pictilisib at a low concentration (1 μM) failed to affect the viability of more than 90% of OS cells. However, this low concentration increased the rate of inhibition of OS cell proliferation by DOX (0.5 μg/ml) by approximately 20% (**Figure 3A**). In addition, flow cytometry showed that pictilisib alone had no effect on apoptosis of OS cells on concentrations ranging from 0 to 2 μM (**Supplemental Figure 1A**). However, we found 1 μM pictilisib increased DOX-mediated apoptosis by at least ten percentage ponits although it did not induce apoptosis of tumor cells over 24 h alone (**Figures 3B, C** **)**.

### Pictilisib Impairs Receptor Activator Of Nuclear Factor Kappa B Ligand-Induced Osteoclast Differentiation and Osteoclastic Bone Resorption *In Vitro*

**Figure 4:**
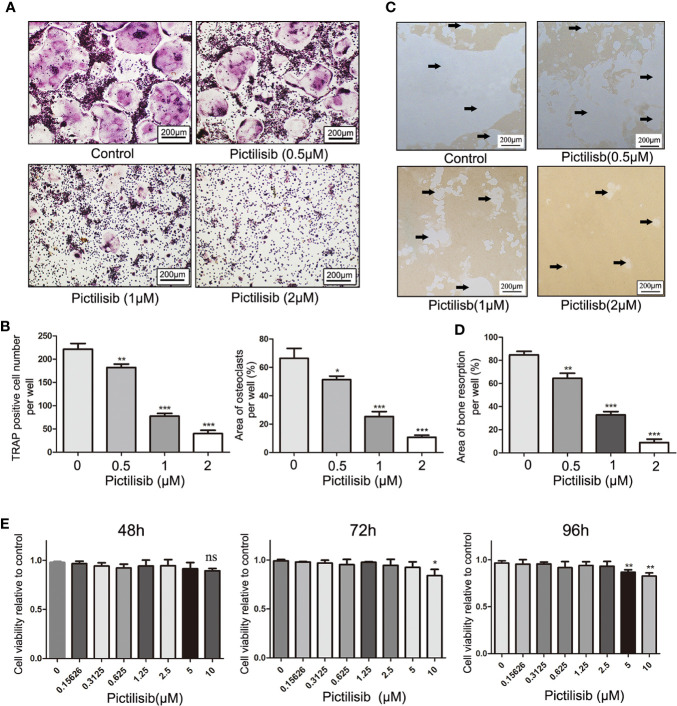
Pictilisib inhibited formation and bone absorption of RANKL-induced osteoclasts *in vitro*. **(A)** Representative images of TRAP-stained multinucleated osteoclasts following treatment with a range of concentrations of pictilisib, 30 ng/ml M-CSF, and 50 ng/ml RANKL for 6 days. **(B)** The number and area of TRAP-positive osteoclasts with at least three nuclei were measured. **(C, D)** BMMs were seeded on a phosphate-coated bone plate in the presence of pictilisib at various concentrations, 30 ng/ml M-CSF, and 50 ng/ml RANKL. At day 7, the resorption pit area was analyzed with ImageJ. **(E)** The proliferation and viability of BMMs treated with a range of concentrations of pictilisib were measured by CCK-8 assay at 48, 72, and 96 h. *P < 0.05, **P < 0.01, ***P < 0.001, ns: no significance.

To examine the effects of pictilisib on osteoclast differentiation, BMMs were induced by 30 ng/ml M-CSF and 50 ng/ml RANKL with various doses of pictilisib (0, 0.5, 1, and 2 μM). The cellular morphology of BMMs was captured under a microscope after TRAP staining on day 7. The control group formed typical well-spread “pancake-shaped” multinucleated osteoclasts with positive TRAP staining. The inhibitory effect of pictilisib was enhanced with increasing concentration. The progression from RANKL-dependent BMMs to mature osteoclasts was totally blocked by 2 μM pictilisib (**Figure 4A**). TRAP-positive cell numbers and areas of osteoclasts per well were calculated and analyzed (**Figure 4B**). Next, a phosphate-coated bone plate was prepared to examine osteoclastic bone resorption. In the control groups without pictilisib, osteoclasts extensively eroded the calcium coating of the bone-mimicking plates. However, the bone absorption function was attenuated by pictilisib in a dose-dependent manner. With 2 μM pictilisib, bone resorption was almost completely abolished in osteoclasts (**Figures 4C, D** **)**. Overall, the results demonstrated that pictilisib could inhibit RANKL-induced osteoclast differentiation and osteoclastic bone resorption *in vitro*. In addition, a CCK-8 assay was performed to evaluate the cell viability of BMMs cultured with pictilisib at a range of concentrations. Pictilisib exhibited no cytotoxicity to BMMs at concentrations below 10 µM for 48, 72, and 96 h (**Figure 4E**).

### Pictilisib Inhibits AKT Phosphorylation and Its Downstream Effectors

**Figure 5:**
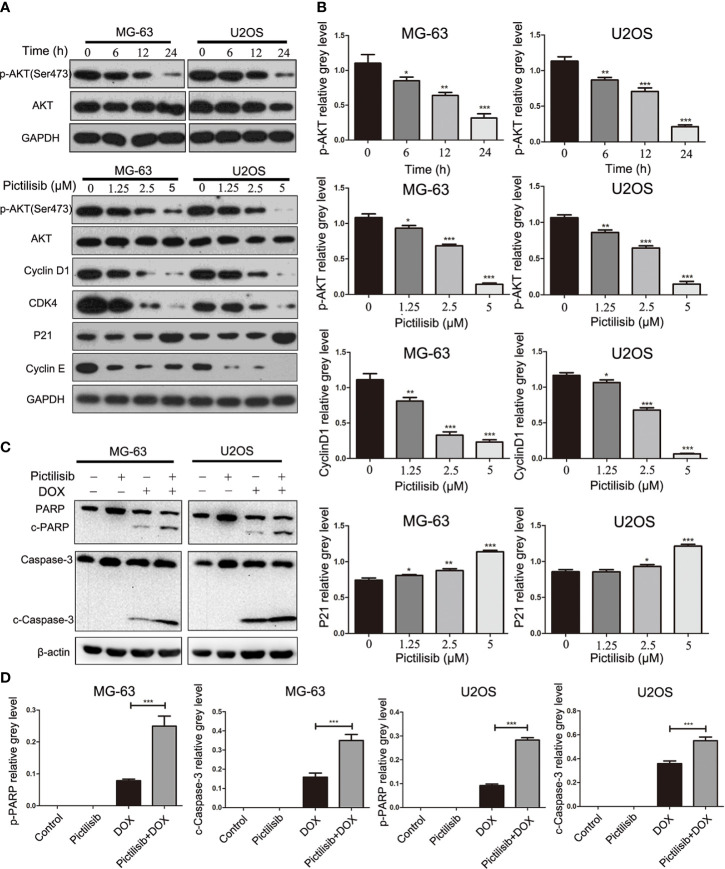
Pictilisib inhibited the phosphorylation of AKT and downstream signaling pathways. **(A)** Western blot analysis was performed to detect total AKT and phosphorylation of AKT in OS cells treated with 2.5μM pictilisib at 0, 6, 12, and 24 h, and with different pictilisib concentrations (0, 1.25, 2.5, and 5 μM) over 24 h. Proteins related to regulation of the cell cycle were detected by western blotting; these included cyclin D1, CDK4, P21, and cyclin E1. **(B)** Quantitative densitometric analysis of p-AKT compared with t-AKT; cyclin D1 and P21 were normalized to GAPDH. **(C)** Total protein extracted from cells treated with 1 μM pictilisib with or without 0.5 μg/ml DOX for 24 h. Apoptosis was evaluated by detection of cleaved PARP and caspase-3 by western blotting. **(D)** Quantitative densitometric analysis of p-AKT compared with t-AKT; cyclin D1 and P21 were normalized to *β*-actin *P < 0.05, **P < 0.01, ***P < 0.001.

Initially, pictilisib was designed to suppress tumor proliferation and overcome resistance to antiestrogen therapy in breast cancer *via* inhibition of all four class I PI3K isoforms (20). The most important downstream effector of PI3K activation is thought to be AKT. Thus, we detected p-AKT expression levels in OS cell lines after treatment with pictilisib. The western blotting results indicated that AKT activation, as evidenced by a reduction in phosphorylation at Ser-473, was inhibited by pictilisib in a dose- and time-dependent manner. Next, we analyzed the expression of downstream genes related to the cell cycle and apoptosis. cyclin D1, cyclin E, and CDK4 were significantly down-regulated in the pictilisib groups compared with the negative control groups (**Figures 5A, B** **)**. These genes encode key proteins involved in the G0/G1-S transition of the cell cycle. There were no significant changes in the expression of cleaved poly(ADP ribose) polymerase (PARP) and cleaved caspase-3 over a range of concentrations of pictilisib (**Supplemental Figure 1C**), but the addition of pictilisib enhanced their expression (**Figures 5C, D** **)**. Together, these results demonstrate that pictilisib inhibited the proliferation of OS cell lines *via* inducing cell cycle arrest at G0/G1 phase and increased their sensitivity to DOX.

**Figure 6:**
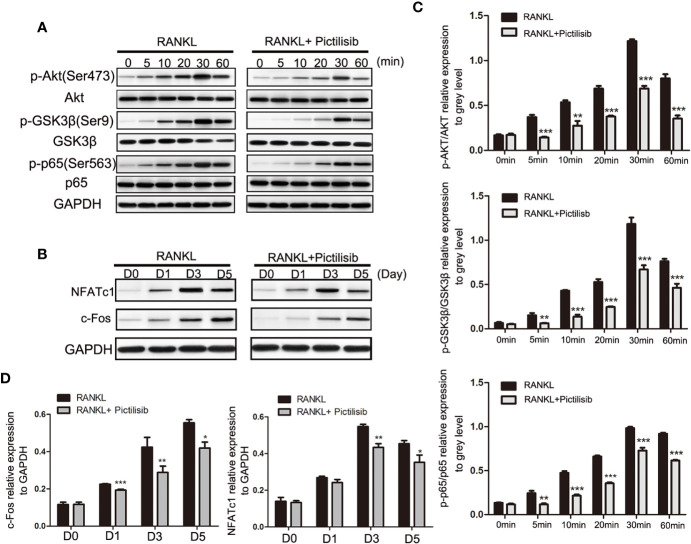
Pictilisib treatment impaired RANKL-induced activation of AKT/GSK3β and NF-*к*B signaling pathways, leading to down-regulation of NFATc1 and c-Fos expression. **(A)** Total cellular proteins were extracted from BMMs stimulated with 50 ng/ml RANKL with or without 2 μM pictilisib for 0, 5, 10, 20, 30, and 60 min and subjected to immunoblotting analysis against total and phosphorylated forms of AkT, GSK3*β*, and NF-*к*B p65. **(B)** Quantitative densitometric analysis of p-AkT, p-GSK3*β*, and p-p65 compared with total protein, respectively. **(C)** BMMs were cultured with 30 ng/ml M-CSF and 50 ng/ml RANKL with or without 2 μM pictilisib for 0, 1, 3, and 5 days, and protein levels of c-Fos and NFATc1 normalized to that of GAPDH were measured. **(D)** Quantitative densitometric analysis of c-Fos and NFATc1 normalized to GAPDH. *P < 0.05, **P < 0.01, ***P < 0.001.

The AKT/GSK3*β* pathway is known to be associated with regulation of NFATc1 during osteoclast differentiation (23). Our results showed that pictilisib effectively inhibited the expression of p-AKT (Ser473) and p-GSK3*β* (Ser9) compared with their total protein contents in BMMs (**Figures 6A, C** **)**. To confirm that the downstream NF-*κ*B pathway was also inhibited by pictilisib, the expression levels of p65 and p-p65 (Ser563) were measured by western blotting. In control groups, we observed that NF-*κ*B p-65 (Ser563) was rapidly phosphorylated by RANKL stimulation (**Figures 6A, C** **)**. Therefore, the target genes NFATc1 and c-fos were upregulated; these genes encode key transcription factors required for osteoclast differentiation. However, this process was attenuated by the addition of pictilisib (**Figures 6B, D** **)**. Thus, the inhibitory effect of pictilisib on osteoclast differentiation may involve modulation of the AKT/GSK3*β* and NF-*κ*B signaling pathways.

### Pictilisib Enhances the Sensitivity of Osteosarcoma to Doxorubicin in Patient-Derived Xenograft Models

**Figure 7:**
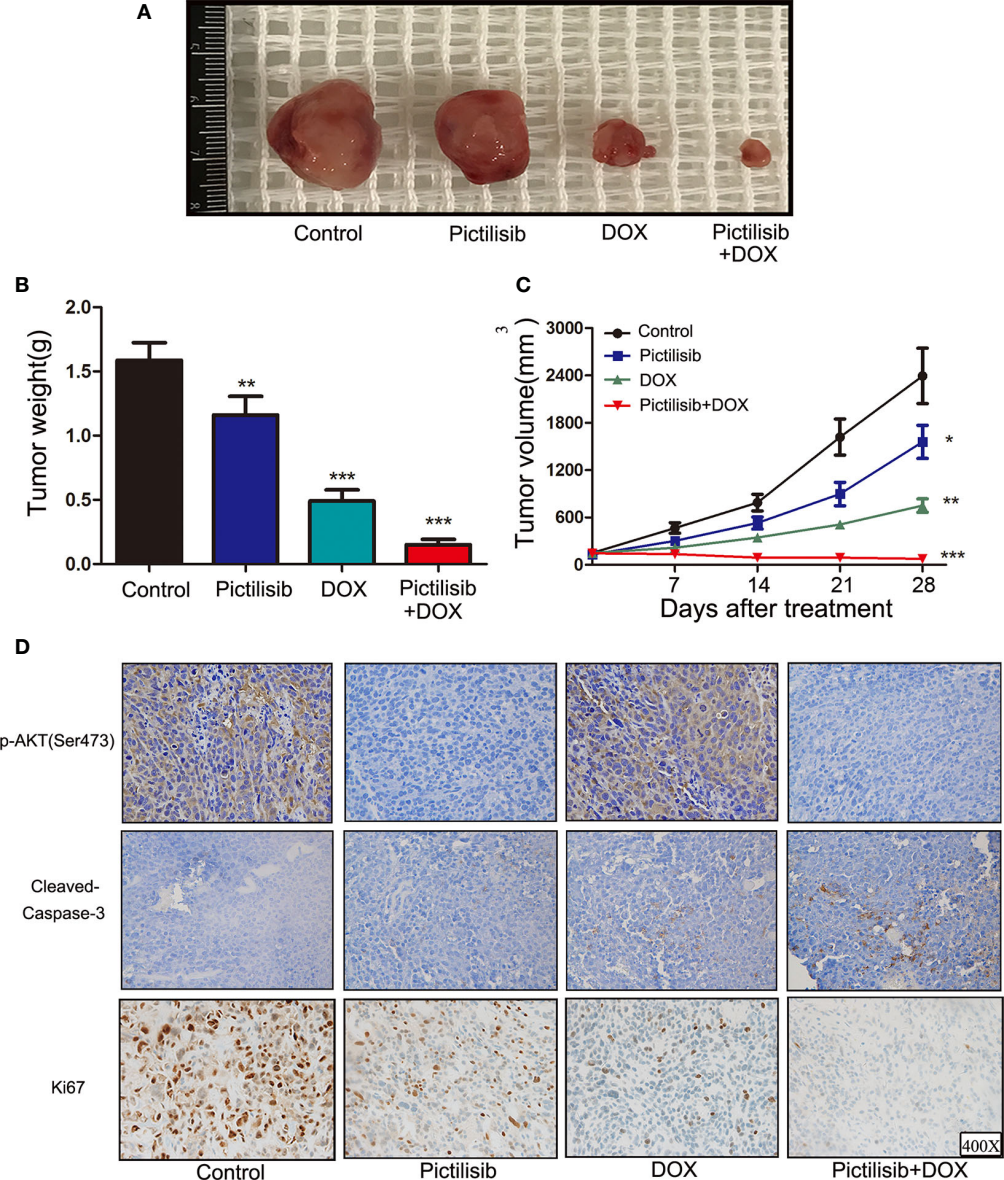
Pictilisib suppressed tumor growth and enhanced sensitivity of chemotherapy in PDX animals. Surgical specimens from a girl with OS were subcutaneously implanted in the abdominal walls of BALB/c-nu mice (n = 5 per group). After 3 weeks, mice were treated with 50 mg/kg pictilisib and/or 2 mg/kg DOX. **(A)** Gross view of tumor specimens from different treatment groups. **(B)** Tumor volumes were calculated once a week following a standard formula: (length × width^2^)/2. **(C)** Tumor sections were weighed on day 28. **(D)** IHC and ki-67 staining were used to measure p-AKT (Ser473) and cleaved-caspase3 (c-caspase3) expression and the degree of proliferation in samples from different groups. *P < 0.05, **P < 0.01, ***P < 0.001.

In recent years, PDX technology has offered more accurate and reliable preclinical models of cancer. Tumor fragments from established models were subcutaneously implanted in the dorsal flanks of BALB/c nude mice. A low dose of pictilisib (50 mg/kg) alone failed to significantly suppress tumor growth compared with the control, but 50 mg/kg pictilisib combined with 2 mg/kg DOX effectively reduced tumor volumes to the size smaller than the initial one over a period of 28 days (**Figures 7A, B** **)**. Tumor weight following treatment with the combination of drugs were lower than those following treatment with either pictilisib or DOX alone (**Figure 7C**). Next, we determined the protein levels of p-AKT (Ser475) and cleaved caspase-3 (Asp175) to test whether pictilisib could enhance the antitumor effects of DOX by downregulating phosphorylation of AKT and upregulating cleaved caspase-3 (Asp175). The results from the PDX model showed that 50 mg/kg pictilisib combined with 2 mg/kg DOX inhibited almost phosphorylation of AKT and significantly elevated the expression of cleaved caspase-3 protein. In addition, Ki-67 staining demonstrated that this combination of pictilisib and DOX reduced the cell population in the proliferative phase compared with single drug treatment (**Figure 7D**). Finally, sections of organs including heart, liver, and kidney were stained with H&E to examine their morphology and assess the toxic effects of treatment (**Supplemental Figure 2A**).

### Pictilisib Slows Tumor Growth in an Orthotopic Model and Reduces Tumor-Induced Osteolysis

**Figure 8:**
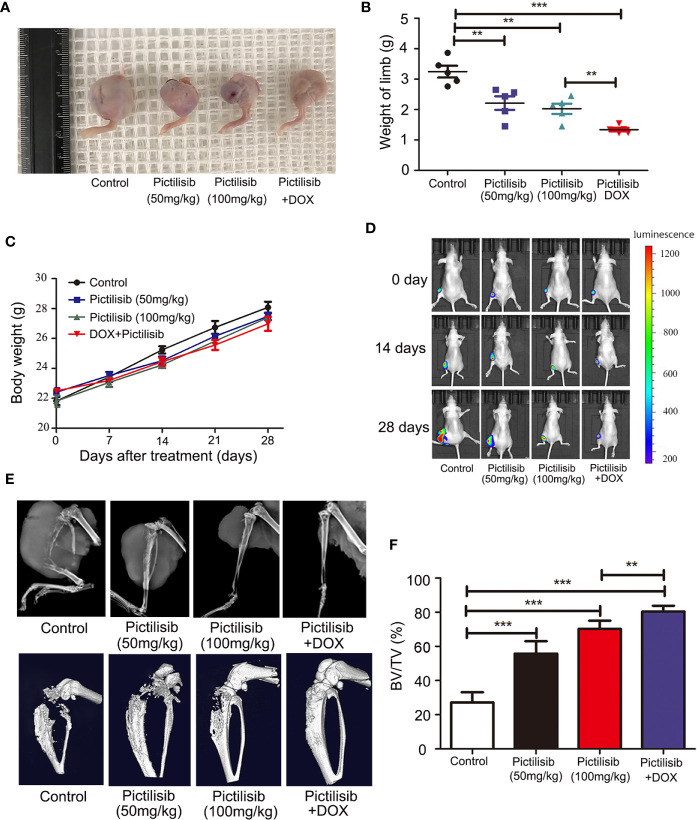
Pictilisib inhibited the growth of tumors and OS-induced osteolysis in orthotopic mouse models. 143B cells expressing luciferase were injected into the backs of mice, then 1 × 1 mm pieces from tumors were embedded into a site adjacent to the left proximal tibia. Mice were treated with PBS, 50 mg/kg pictilisib, 100 mg/kg pictilisib, or 50 mg/kg pictilisib + 2 mg/kg DOX for 28 days. **(A)** Representative photos of left posterior limb from each group after sacrifice. **(B)** The weights of left legs with tumors were measured on day 28. **(C)** Changes in weight of each group on average after treatment. **(D)** Tumor growth was monitored by measuring luciferase activity in tumor-bearing mice after treatment on days 0, 14 and 28. **(E)** Tumor-induced osteolysis was measured by X-ray and micro-CT on day 28. Typical X-ray and three-dimensional reconstructed images are shown. **(F)** Quantitative analyses of BV/TV in the region of interest. **P < 0.01, ***P < 0.001.

The therapeutic value of pictilisib was studied in an orthotopic mouse model of OS using the 143B-luc cell line. Tumors of 1 × 1 × 1 mm^3^ in size were implanted on the surfaces of the left tibiae of mice. The mice received treatment of 50 or 100 mg/kg pictilisib and/or 2 mg/kg DOX for 4 weeks. Mice treated with the low or high dose of pictilisib showed slower tumor growth compared with control mice. Besides, the combination treatment with pictilisib and DOX significantly reduced tumor sizes (**Figures 8A, B** **)**. Nevertheless, weight of mice was not significantly affected by pictilisib (**Figure 8C**). Luciferase imaging was performed on days 0, 14, and 28 to observe changes in tumor size (**Figure 8D**). Next, the role of pictilisib in tumor-induced osteolysis was assessed by X-ray analysis and high-resolution micro-CT. In control groups, pathological fracture was induced by severe osteolysis. Treatment with pictilisib at a dose of 100mg/kg reduced OS-mediated bone destruction (**Figure 8E**). Quantitative analyses of bone morphometric parameters demonstrated that pictilisib markedly increased BV/TV in the region of interest (**Figure 8F**).

## Discussion

OS is the most common primary malignant bone tumor and occurs mainly in children and adolescents. The introduction of neoadjuvant chemotherapy in the 1970s led to a dramatic increase in overall survival rates from 20 to 50–70%, but many novel therapeutic approaches developed over the past 40 years have failed to achieve higher cure rates (3). Targeted therapies have achieved great success in some malignant bone and soft-tissue sarcomas (24–26), there is now a critical need to develop new targeted drugs to improve clinical outcomes in OS. Targeting PI3K represents a potential strategy to treat OS (27). Here, we found that 75.9% patients who provided biopsy samples were p-AKT positive; this was significantly higher than the 32.2% of patients after multi-drug chemotherapy. Our findings confirmed that positive expression of p-AKT after chemotherapy was associated with lower TCNR (<90%). This indicated that neoadjuvant chemotherapy effectively eliminated the majority of tumor cells owing to strong proliferation signals in most patients. The remaining p-AKT-positive patients showed a poor response to chemotherapy. TCNR following chemotherapy is well known to have a strong influence on patient survival and prognosis. Thus, molecular inhibition of the PI3K/AKT pathway may be an alternative strategy for treatment of patients with positive expression of p-AKT before or after surgery.

Pictilisib, a selective and potent oral inhibitor of class I PI3K, was initially developed for the treatment of advanced breast cancer in combination with bevacizumab, trastuzumab, or letrozole (20). Here, we found that the proliferation of several different OS cell lines could be inhibited by pictilisib to varying degrees; it had the most powerful effect on MG-63 cells, whereas 143B cells were slightly inhibited. In another study, 143B cells harboring an oncogenic KRAS transformation did not show any response to MK-2206, an AKT inhibitor, at concentrations below 5 μM. Kuijjer proposed that insensitivity to inhibition of the AKT pathway was mainly caused by activation of the Ras/Raf/ERK pathway, based on differences in kinome profiles of 143B cells treated with or without MK-2206 (8). Next, we demonstrated that the inhibition of OS depended on cell cycle arrest in G0/G phase *in vitro*. Cyclin D1 is known to have a key role in G1 to S phase transition and to act as a downstream target of p-AKT. In addition, overexpression and amplification of cyclin D1 have been detected in 22 and 4% of OS samples, respectively (28). However, according to the flow cytometry and western blotting analyses, pictilisib did not affect apoptosis rates in OS cell lines. Another study showed that NVP-BEZ235, a dual PI3K/mTOR inhibitor, was also unable to induce apoptosis of OC cell lines (29). This may have been because of the activation of the MEK/Erk pathway (30). Pictilisib showed a stronger inhibitory effect when combined with DOX, indicating that pictilisib may improve sensitivity to chemotherapy by reducing the occurrence of drug-resistant cells.

**Figure 9:**
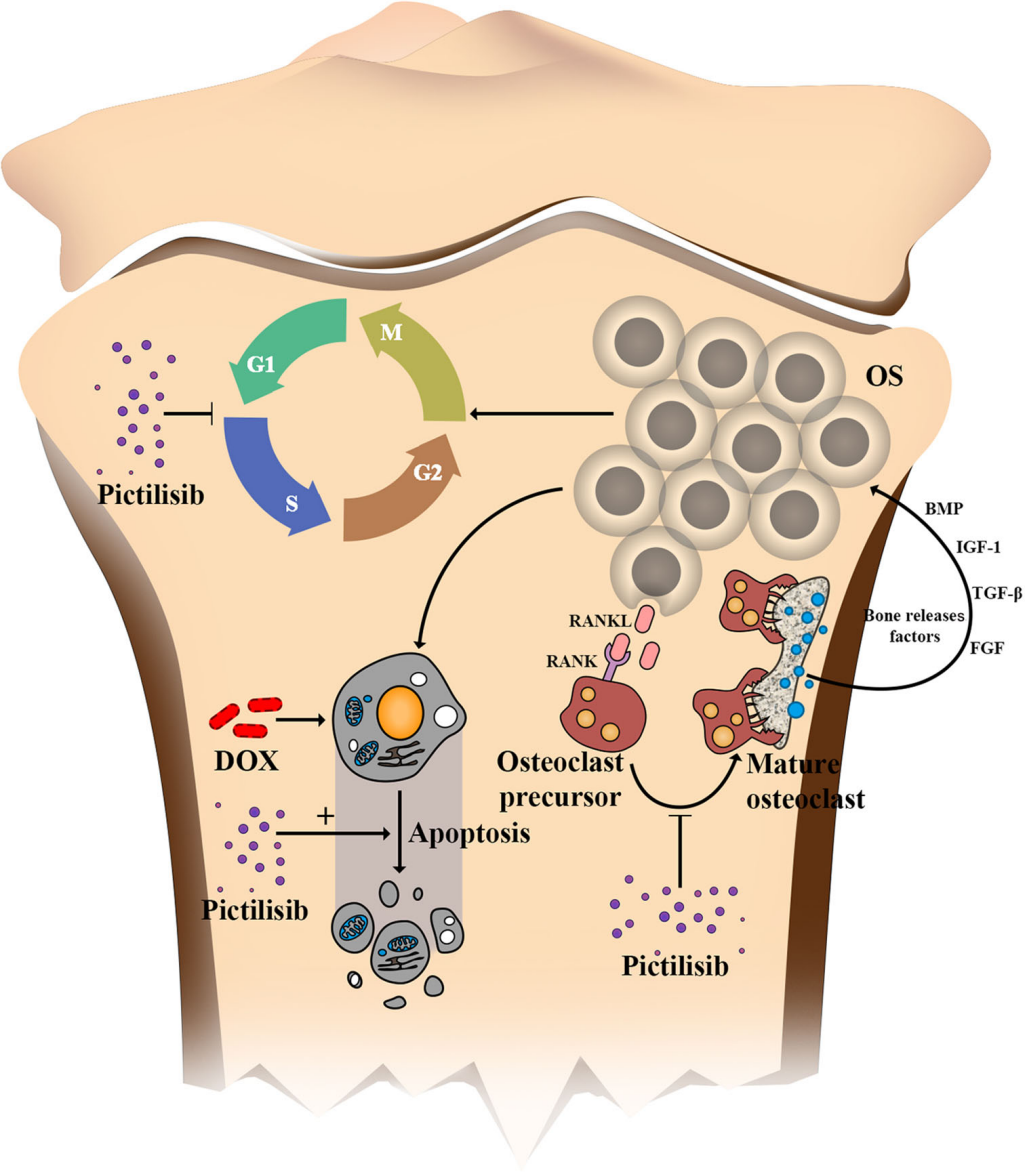
Schematic of the effects of pictilisib on OS cells and osteoclasts in the tumor microenvironment. In brief, pictilisib inhibited proliferation of OS *via* cell cycle arrest rather than induction of cell apoptosis. In addition, pictilisib enhanced the sensitivity of OS cells to DOX. Further, tumor-induced osteolysis was effectively prevented by pictilisib.

Further, the “seed and soil” theory of cancer is widely accepted, and the OS microenvironment is composed of bone matrix including osteoclasts, osteoblastomas, fibrocytes, and endothelial cells. Previous studies have suggested that osteoclasts provide “nutrition” for OS but increase the risk of lung metastasis if they are inhibited at a late stage (31). In our research, TRAP-positive osteoclasts were efficiently inhibited by pictilisib *in vitro via* down-regulation of the AKT/GSK3*β*/NFATc1 and NF-*κ*B pathways. We tested the efficiency of pictilisib in inhibition of tumor growth and tumor-induced osteolysis in orthotopic mice models. In other studies, zoledronic acid alone or combined with ifosfamide showed good results (32, 33). However, we failed to observe lung metastasis in any group, so it was unclear whether pictilisib had an effect on lung metastasis (**Supplemental Figure 2B**). In our study, lipopolysaccharide (LPS)-induced osteolysis was also inhibited by pictilisib in a mouse calvaria model (**Supplemental Figures 3A–F**). Inflammation-induced osteolysis and tumor-mediated bone destruction have shared downstream pathways involving interaction between RANKL and RANK in activation of osteoclasts (**Figure 9**). These include the AKT/GSK3*β*/NFATc1 and NF-*κ*B pathways, which are the main regulators of differentiation and activation of osteoclasts (34, 35). A limitation of pictilisib is its lack of a cytotoxic effect; thus, it is necessary to combine it with other cytotoxic drugs.

Overall, our findings indicate that pictilisib combined with standard chemotherapy may be an efficient treatment for patients with positive p-AKT (Ser473) expression.

## Data Availability Statement

The original contributions presented in the study are included in the article/**Supplementary Material**; further inquiries can be directed to the corresponding authors.

## Ethics Statement

The studies involving human participants were reviewed and approved by the Ethics Committee of Shanghai Sixth People’s Hospital. Written informed consent to participate in this study was provided by the participants’ legal guardian/next of kin.

## Author Contributions

YD and ZS designed and conceptualized the project. CL, XY, and NX performed the research and collected the data. ZZ performed the data statistical analysis. CL drafted the manuscript. All authors contributed to the article and approved the submitted version.

## Funding

This research was supported by grants from the Interdisciplinary Research Fund of Translational Medicine from Shanghai Jiao Tong University (ZH2018QNB06).

## Conflict of Interest

The authors declare that the research was conducted in the absence of any commercial or financial relationships that could be construed as a potential conflict of interest.

## Supplementary Material

Supplementary Figure 1 Low-dose pictilisib failed to induce apoptosis of OS cells (MG-63, U2OS). **(A, B)** Apoptotic cells induced with a range of concentrations of pictilisib were detected by flow cytometry. Proportions of apoptotic cells were compared among the four groups. **(C)** Expression of cleaved PARP and caspase-3 was evaluated by western blotting. *P < 0.05. ns, no significance.

Supplementary Figure 2 H&E staining of organs was performed to assess the toxicity of pictilisib in PDX models and lung metastasis in orthotopic models. **(A)** H&E staining of sections of heart, lung and kidney in the four groups. **(B)** H&E staining of sections of lung samples from orthotopic models.

Supplementary Figure 3 Pictilisib reduced osteolysis caused by LPS-induced inflammation *in vivo*. Collagen sponges soaked with PBS (control) or 10 mg/kg LPS were implanted over murine calvarias, and mice received subcutaneous injection of PBS or pictilisib (20 nM or 2 μM) over the sagittal middle suture of the calvaria for 10 days. **(A)** Representative three-dimensional micro-CT reconstructed images of mice calvaria. **(B–E)** Quantitative morphometric analyses of BV/TV (%), number of pores, bone mineral density(BMD) (mg/cc), and porosity as a percentage. **(F)** Representative images of H&E (×50 and ×100) and TRAP (×100 and ×200) staining in control and treatment groups. *P < 0.05, **P < 0.01, ***P < 0.001.
