# Supplementary figures and images for: Pictilisib Enhances the Antitumor Effect of Doxorubicin and Prevents Tumor-Mediated Bone Destruction by Blockade of PI3K/AKT Pathway
Source: Front Oncol. 2021 Feb 15;10:615146. doi: 10.3389/fonc.2020.615146 (PMC7917262; doi:10.3389/fonc.2020.615146)

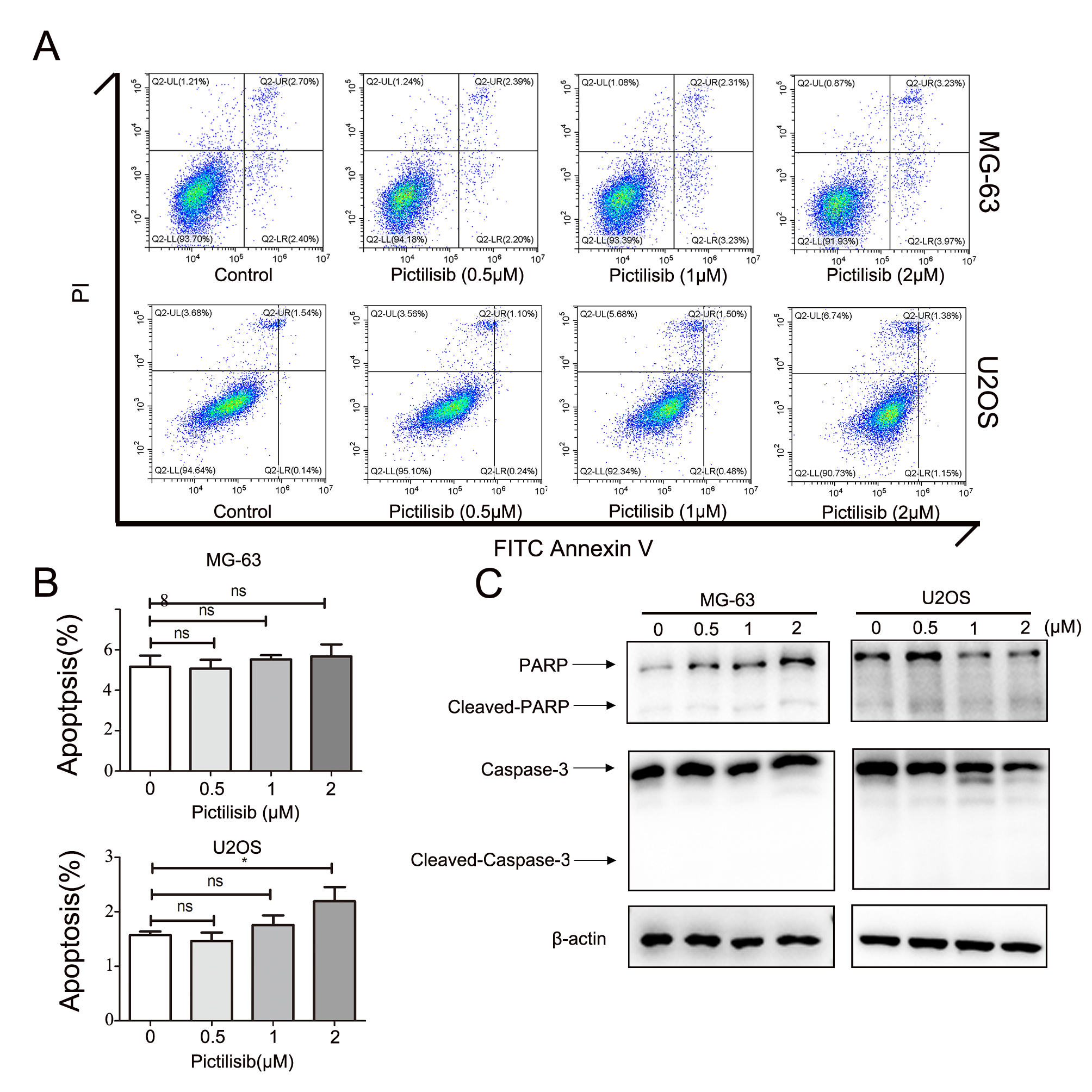

Supplement: Supplementary Figure 1 — Low-dose pictilisib failed to induce apoptosis of OS cells (MG-63, U2OS). (A, B) Apoptotic cells induced with a range of concentrations of pictilisib were detected by flow cytometry. Proportions of apoptotic cells were compared among the four groups. (C) Expression of cleaved PARP and caspase-3 was evaluated by western blotting. *P < 0.05. ns, no significance. [file Image_1.tif]

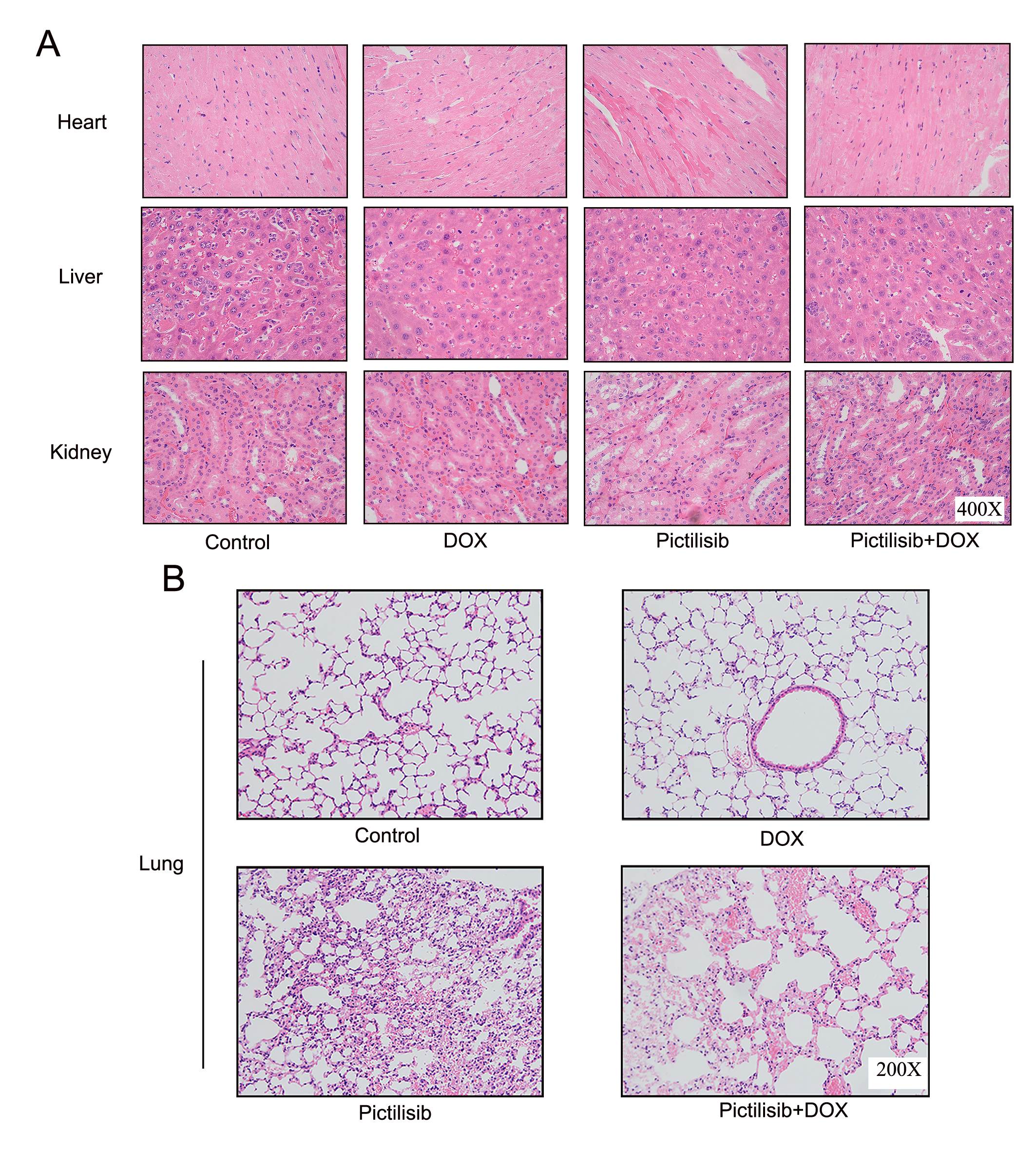

Supplement: Supplementary Figure 2 — H&E staining of organs was performed to assess the toxicity of pictilisib in PDX models and lung metastasis in orthotopic models. (A) H&E staining of sections of heart, lung and kidney in the four groups. (B) H&E staining of sections of lung samples from orthotopic models. [file Image_2.tif]

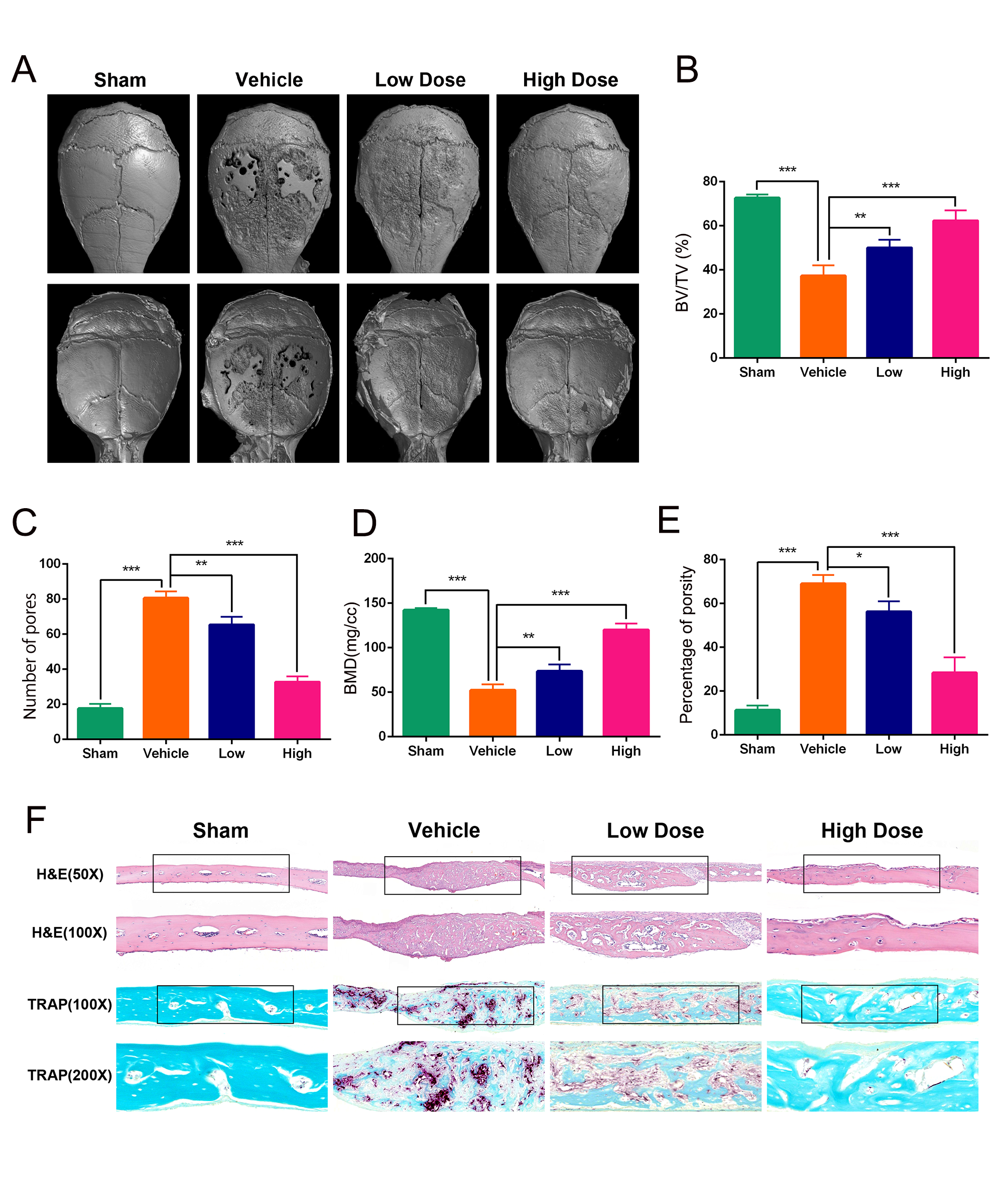

Supplement: Supplementary Figure 3 — Pictilisib reduced osteolysis caused by LPS-induced inflammation in vivo. Collagen sponges soaked with PBS (control) or 10 mg/kg LPS were implanted over murine calvarias, and mice received subcutaneous injection of PBS or pictilisib (20 nM or 2 μM) over the sagittal middle suture of the calvaria for 10 days. (A) Representative three-dimensional micro-CT reconstructed images of mice calvaria. (B–E) Quantitative morphometric analyses of BV/TV (%), number of pores, bone mineral density(BMD) (mg/cc), and porosity as a percentage. (F) Representative images of H&E (×50 and ×100) and TRAP (×100 and ×200) staining in control and treatment groups. *P < 0.05, **P < 0.01, ***P < 0.001. [file Image_3.tif]
