# Supplementary material for: Pictilisib Enhances the Antitumor Effect of Doxorubicin and Prevents Tumor-Mediated Bone Destruction by Blockade of PI3K/AKT Pathway
Source: Front Oncol. 2021 Feb 15;10:615146. doi: 10.3389/fonc.2020.615146 (PMC7917262; doi:10.3389/fonc.2020.615146)
Supplement: Supplementary file 4 [file DataSheet_1.doc]

Supplemental information

The following primary antibodies in WB were used: rabbit monoclonal anti-Akt (#4685, 1:1000, Cell Signaling Technology, USA), rabbit monoclonal anti-phospho-Akt (Ser473) (#4060, 1:1000, Cell Signaling Technology, USA), rabbit monoclonal anti-CyclinD1 (#2978, 1:1000, Cell Signaling Technology, USA), rabbit monoclonal anti-p21 (#2947, 1:1000, Cell Signaling Technology, USA), rabbit monoclonal anti-CDK4 (#12790, 1:1000, Cell Signaling Technology, USA), rabbit monoclonal anti-PARP (#9532, Cell Signaling Technology, USA), rabbit polyclonal anti-Caspase-3 (#9662, Cell Signaling Technology, USA), rabbit monoclonal anti-GSK-3β (#12456, 1:1000, Cell Signaling Technology, USA), rabbit monoclonal anti-phospho-GSK-3β (Ser9) (#5558, 1:1000, Cell Signaling Technology, USA), rabbit monoclonal anti-NF-κB p65 (#8242, 1:1000, Cell Signaling Technology, USA), rabbit monoclonal anti-phospho-NF-κB p65(Ser536) (#3033, 1:1000, Cell Signaling Technology, USA), rabbit monoclonal anti-NFATc1 (#8032, 1:1000, Cell Signaling Technology, USA), rabbit monoclonal anti-c-Fos (#2250, 1:1000, Cell Signaling Technology, USA), rabbit monoclonal anti-GAPDH (#5174, 1:1000, Cell Signaling Technology, USA) antibodies, rabbit monoclonal anti-β-Actin (#5174, 1:1000, Cell Signaling Technology, USA). It is noteworthy that rabbit anti-human cleaved caspase-3 (Asp175) (#9661, Cell Signaling Technology, USA) antibody was used in IHC.
